# Current Treatment and Outcomes Benchmark for Locally Advanced or Metastatic Urothelial Cancer From a Large UK-Based Single Centre

**DOI:** 10.3389/fonc.2020.00167

**Published:** 2020-02-20

**Authors:** Sue Cheeseman, Matthew Thompson, Will Sopwith, Paul Godden, Divyagiri Seshagiri, Lola Adedokun, Kieran Zucker, Sunjay Jain, Sanjeev Kotwal, Stephen Prescott, Ann Henry, Joji Joseph, Sameer Chilka, Jo-An Roulson, Michael Weston, Simon Burbidge, Simon Brown, Satinder Jagdev, Christy Ralph, Geoff Hall, Naveen S. Vasudev

**Affiliations:** ^1^Leeds Cancer Centre, St James's University Hospital, Leeds, United Kingdom; ^2^IQVIA, London, United Kingdom; ^3^Janssen Pharmaceuticals, Wycombe, United Kingdom; ^4^Bradford Royal Infirmary, Bradford, United Kingdom; ^5^School of Medicine, University of Leeds, Leeds, United Kingdom

**Keywords:** carboplatin, cisplatin, real-world, chemotherapy, urothelial cancer

## Abstract

**Objectives:** To characterize treatment patterns and survival outcomes for patients with locally advanced or metastatic malignancy of the urothelial tract during a period immediately preceding the widespread use of immune checkpoint inhibitors in the UK.

**Patients and Methods:** We retrospectively examined the electronic case notes of patients attending the Leeds Cancer Center, UK with locally advanced or metastatic urothelial carcinoma, receiving chemotherapy between January 2003 and March 2017. Patient characteristics, treatment patterns, and outcomes were collected. Summary and descriptive statistics were calculated for categorical and continuous variables as appropriate. The Kaplan–Meier method was used to estimate median survival and Cox regression proportional hazards model was used to explore relationships between clinical variables and outcome.

**Results:** Two hundred and sixteen patients made up the study cohort, with a median age of 66 years (range: 35–83) and 72.7% being male. First-line treatment consisted of either a cisplatin- (44%) or carboplatin-based regimen (48%) in the majority of patients. Twenty seven percent of patients received a second-line of treatment (most commonly single-agent paclitaxel) following a first-line platinum containing regimen. Grade 4 neutropenia was observed in 19 and 27% of those treated with a first-line cisplatin- and carboplatin-based regimen, respectively. The median overall survival (mOS) of the study cohort was estimated to be 16.2 months (IQR: 10.6–28.3 months). Receipt by patients of cisplatin-based chemotherapy was associated with a longer mOS and this association persisted when survival analysis was adjusted for age, sex, performance status and presence of distant metastases.

**Conclusions:** This study provides a useful benchmark for outcomes achieved in a real-world setting for patients with locally advanced or metastatic UC treated with chemotherapy in the immediate pre-immunotherapy era.

## Introduction

Malignant neoplasms of the urinary tract include cancers of the bladder, renal pelvis, ureter and urethra, but are increasingly recognized as a single site in international coding schemes when diagnosed at the same time (1). The majority of patients diagnosed with these malignancies have urothelial cancer (UC) (also known as transitional cell carcinoma), but rarer types are also recognized, such as squamous cancers, adenocarcinomas, and neuroendocrine tumors. The most common site, bladder cancer, is the ninth most common cancer overall in the UK and the sixth most common in men (2). Recognized risk-factors for the development of bladder cancers include tobacco smoking, chemical exposure and chronic urinary tract infections (3).

In 2014, ~25% of patients with bladder cancer in England had locally advanced or metastatic disease at the time of presentation (4). Internationally, platinum-based chemotherapy has formed the standard of care for such patients at first-line for more than three decades (5–7). Current European Association of Urology guidance estimates 50% of patients with UC are not sufficiently fit to tolerate cisplatin-based therapy (8). Carboplatin-based regimens represent an alternative first-line treatment for those with poor renal function or poor performance status (PS). However, outcomes with carboplatin are recognized to be less favorable (7). Second-line treatment options are limited and no standard of care has been established, with paclitaxel, docetaxel, and vinflunine all variably employed. Vinflunine is not currently approved for use in the UK (9).

With the recent introduction of checkpoint inhibitors (CPI) targeting programmed cell death protein-1 or its ligand (PD-1/PD-L1), the treatment landscape for patients with metastatic UC (mUC) has been transformed. In the first-line setting, immunotherapy now forms a new standard of care in patients who are ineligible for cisplatin and whose tumors express PD-L1 (10, 11). In the second-line setting, following platinum-failure, pembrolizumab has been shown to increase overall survival in comparison to existing treatment options (12). Studies examining first-line combination anti-PD1/PDL-1 plus cytotoxic T-lymphocyte-associated antigen-4 (CTLA-4) are also underway (13) that may further alter the therapeutic pathway.

Despite treatment, prognosis is generally poor for patients with metastatic disease; median survival between 15 and 18 months is reported in clinical trials (14). However, non-concordance between clinical trials and real-world practice is well-recognized (15) and there is little published information on real-world outcomes for patients with locally advanced or metastatic UC in the UK. As the treatment landscape changes, gaining a fuller understanding of current clinical practice and patient outcomes sets an important benchmark against which the real-world impact of novel therapies can be evaluated.

The aim of this study was to characterize the existing population of patients treated within a large regional cancer center with such malignancies in the period immediately preceding the introduction of CPI into routine clinical practice. Study objectives included defining the baseline demographic and clinical characteristics of patients at first presentation, describing treatments received, summarizing survival, and quantifying the incidence of selected adverse events.

## Patients and Methods

### Study Design

This study was conducted using a retrospective longitudinal cohort design, with secondary use of electronic medical records data. Eligible patients were identified from a major regional NHS cancer center in the UK between January 2003 and March 2017 and followed-up from first presentation (index date) until February 2018 to observe survival outcomes and selected adverse effects.

### Setting

The Leeds Cancer Center (LCC) serves a metropolitan catchment area of over 850,000 people for secondary care and 2.7 million as a regional cancer center. LCC is hosted by Leeds Teaching Hospitals NHS Trust (LTHT). Patient data including all demographics, cancer diagnoses and staging and anti-cancer therapy are managed in patient pathway manager (PPM), an in house LTHT EMR developed and brought into routine clinical use in 2003.

This study was conducted by the Real-world Evidence Alliance Leeds (REAL), a collaboration between LCC, Leeds Institute for Data Analytics and IQVIA™. REAL accesses continually updated patient data stored in PPM and unstructured data within patient notes was additionally reviewed by a consultant medical oncologist both to maximize data completeness and to enable derivation of outcome variables. REAL studies are conducted on-site within LTHT under the strict legal framework governing access to and use of personal information in the NHS (16). The study was completed with UK Health Research Authority formal approval; the need for ethics approval was waived.

### Participants

Eligible patients for inclusion were those adult patients (≥18 years old) with a confirmed incident diagnosis of advanced or metastatic malignancy of the urothelial tract and recorded treatment with chemotherapy between January 2003 and March 2017. Advanced or mUC was defined according to the appropriate International Classification of Disease (ICD)-10 codes (C65–C68) and tumor, nodes and metastasis (TNM) staging (T4b, N0, M0, or Tany, N1-3, M0, or Tany, Nany, M1). Patients were excluded if any one or more of the following applied: incomplete treatment records (e.g., where an incident diagnosis was made in a center outside the scope of LCC); periods of early treatment were missing; incomplete TNM staging; significant other malignancies were present; and/or tumor morphology other than urothelial carcinoma.

### Data Variables

For this study, total person-time was defined by diagnosis index date (first diagnosis of locally advanced or mUC) or treatment index date (date of first treatment), censor date (if lost to follow-up) or end of study period (February 2018).

Unless otherwise stated, patient and clinical characteristics were as recorded in PPM at diagnosis index date or the date closest to (and following) index date. Smoking status was not well recorded in PPM and therefore extracted from unstructured patient notes during the process of clinical review. Performance status (PS) was as defined by the World Health Organization or Eastern Cooperative Oncology Group (ECOG) scheme (17). TNM staging followed the Union for Cancer Control (UICC)/American Joint Committee on Cancer (AJCC) TNM classification (7th edition) (18). Morphology was defined in accordance with ICD-10- morphology codes and the study cohort included patients with transitional cell carcinomas (ICD10 code M8120/0-3).

Treatment was reported by line of therapy and regimen—all regimens of chemotherapy recorded in PPM were reported. Treatment with radiotherapy was reported by treatment intent. Line of therapy was designated as treatment following index date and sequential line of therapy derived during review of clinical notes. Treatment duration and time between treatments was defined according to respective start and end dates of line of therapy. Surgical treatment was not collected.

Summary and descriptive statistics were calculated for categorical and continuous variables as appropriate.

### Patient Outcomes

Tumor response to treatment was derived from radiology reports recorded in PPM by the treating clinician using variables “complete response,” “partial response,” “stable disease,” or “progressive disease.” Response was not further reviewed or confirmed for the study. Overall Survival (OS) was defined as time since index date until date of death due to any cause. Confirmed date of death was used, following monthly reconciliation of PPM with Office for National Statistics death certifications. Patients without any record of death during the study period were censored accordingly. Progression Free Survival (PFS) was defined as time since index date until the first disease progression date. Patients without any record of disease progression during the study period were censored accordingly. Overall response rate (ORR) was defined as the proportion of patients in a particular sub-group experiencing a complete or partial response following treatment. Disease control rate (DCR) was defined as the proportion of patients in a particular sub-group experiencing an overall response (as above) or stable disease.

### Data Analysis

Summary statistics were calculated for the demographic and treatment pattern characteristics of patients within the final evaluable cohort. Continuous variables were described by the mean, standard deviation, median, 25th and 75th percentiles, minimum and maximum. Categorical variables were described by the number and percentage of patients in each category. Differences between categorical variables were tested using Pearson χ^2^ test, and differences between continuous variables tested by using parametric two sample *t*-tests, where appropriate.

OS from index date (diagnosis or treatment as appropriate) were estimated using the Kaplan Meier survival methodology and depicted graphically by Kaplan-Meier curves, with number of at-risk patients at particular time-points tabulated below the curves. Censored patients are indicated graphically. Median OS was reported, including 25th and 75th percentiles. A Cox proportional hazards regression analysis was also conducted to calculate adjusted hazard ratios (HR) with 95% confidence intervals (95% CI) for identified predictor variables. Model diagnostics assessed the robustness of the model.

## Results

### Patient Characteristics

Of 483 patients identified, 199 were excluded based on TNM staging and a further 68 were excluded based on treatment record or other exclusion criteria. The study cohort consisted of 216 patients, diagnosed with advanced or metastatic urothelial carcinoma between 2003 and 2017. Summary characteristics of the evaluable cohort are shown in Table 1. The study cohort was 72.7% male with a median age of 66 years (IQR: 59–72 years; range: 35–83 years). The most common primary tumor site was in the bladder (77.3%; *n* = 167) with ureter or renal pelvis accounting for almost all others (20.8%; *n* = 45). Where smoking status was recorded (*n* = 194), the majority were ex-smokers (43.1%; *n* = 93) or current smokers (26.4%; *n* = 57). Over a third of patients had a BMI status below 25 kg/m^2^ (38.4%; *n* = 83). Where ECOG PS was reported (*n* = 168), 85.1% (*n* = 143) had a score <2. Over half of the study cohort (56.9%, *n* = 123) had distant metastases (M1) at index date, the most common sites being lymph nodes (31.0%; *n* = 67), pulmonary (15.3%; *n* = 33), and bone (14.8%; *n* = 32).

**Table 1:** Patient Characteristics of study cohort.

**Patient characteristic (% all)**		**Study cohort (*N =* 216)**
Male		157 (72.7%)
Age	Median (IQR) Mean (SD)	66 (59,72) 65.0 (9.4)
Tumor site	Bladder	167 (77.3%)
	Ureter	31 (14.4%)
	Renal pelvis	14 (6.5%)
	Urethra	<6
TNM at index	T4b,N0,M0	6 (2.8%)
	Tany,N1-3,M0	87 (40.3%)
	Tany,Nany,M1	123 (56.9%)
Smoking status	Current smoker	57 (26.4%)
	Ex-smoker	93 (43.1%)
	Never smoked	18 (8.3%)
	Non-smoker (history unknown)	26 (12.0%)
	Smoking status NK	22 (10.2%)
ECOG^a^	0	66 (30.6%)
	1	77 (35.6%)
	2	24 (11.1%)
	3	<6
	NK	48 (22.2%)
BMI^b^	Mean (SD)	26.54 (4.73)
	<25	83 (38.4%)
	25–29.9	84 (38.9%)
	30+	47 (21.8%)

a *ECOG recorded for patients at the first point of receiving treatment with chemotherapy following index date*.

b *Body mass index: measures of 25 and above are considered to exceed healthy weight, with 30 being the threshold for obesity in the UK (19). Data was missing for 2 patients*.

### First-line Treatment Patterns

In addition to receiving systemic treatment at least once following index date, 44.9% (*n* = 97) of patients in the study cohort also received radiotherapy. The majority of patients receiving radiotherapy (75%; *n* = 73) were treated with palliative intent, but 24 (24.7%) were treated with radical radiotherapy, either in addition to or instead of palliative treatment. Cisplatin/gemcitabine and carboplatin/gemcitabine were the most commonly received chemotherapy regimens at any point following index date [41.7% (*n* = 90) and 40.7% (*n* = 88), respectively] but patients also received a range of other platinum- and non-platinum-based therapies (Table 2).

**Table 2:** Treatment regimens at any time throughout study (all lines of therapy).

**Chemotherapy category**	**Regimen**	**Study cohort (*N* = 216)**
Cisplatin-based	Cisplatin/gemcitabine	90 (41.7%)
	Accelerated MVAC^a^	7 (3.2%)
	Gemcitabine/cisplatin/sunitinib	<6
Carboplatin—based	Carboplatin/gemcitabine	88 (40.7%)
	Carboplatin MV^b^	27 (12.5%)
	Carboplatin/gemcitabine (TOUCAN trial)	9 (4.2%)
	Carboplatin/paclitaxel	8 (3.7%)
Non-platinum	Gemcitabine	13 (6.0%)
	Paclitaxel	32 (14.8%)
Other^c^	Carboplatin/etoposide	6 (2.8%)
	CAV^d^	<6

a *MVAC, Methotrexate/vinblastine/doxorubicin/cisplatin*.

b *MV, Methotrexate/vinblastine*.

c *Other therapies include treatments for small cell carcinoma UC*.

d *CAV, Cyclophosphamide/doxorubicin/vincristine*.

At first-line of treatment, 44.4% (*n* = 96) of patients were treated with a cisplatin-based regimen (“cisplatin sub-cohort”), 48.1% (*n* = 104) with a carboplatin-based regimen (“carboplatin sub-cohort”) and 5.1% (*n* = 11) with a non-platinum single agent (either paclitaxel or gemcitabine) (“non-platinum sub-cohort”). No patients in the non-platinum sub-cohort were diagnosed or first treated later than 2010. The remaining patients were treated at first-line with therapies generally used for small cell disease or experimental regimens and are not included in subsequent analysis. There was a small number of patients (<6) who were started on cisplatin-based chemotherapy but switched to carboplatin during the first-line of therapy and these are included in the cisplatin-based sub-cohort.

Where baseline characteristic data were stratified by platinum regimen treatment type, there was no difference in the distribution of sex, smoking status, or tumor site (Table 3). Patients within the cisplatin sub-cohort were younger and more likely to have a BMI below 25 kg/m^2^ than those in the carboplatin sub-cohort. Patients within the carboplatin sub-cohort were more likely to have a PS of at least 2 and to have metastatic disease at index date than those in the cisplatin sub-cohort. The distribution of distant metastases sites was similar between the two sub-cohorts, with metastases of lymph nodes most commonly recorded. Patients in the non-platinum sub-cohort were older (median 72, range 69–83) and more likely to have PS of at least 2 (data not shown). Response rates to chemotherapy, overall and by first-line regimen, are shown in Table 4. Patients in the cisplatin sub-cohort were more likely than the carboplatin sub-cohort to have a complete response [χ^2^, *df* (1), *p* = 0.03] and less likely to have progressive disease [χ^2^, *df* (1), *p* < 0.01].

**Table 3:** Characteristics of patients treated with a first-line platinum-based agent following index date, by regimen type.

**Patient characteristic (% sub-cohort)**		**Cisplatin sub-cohort (*N* = 96)**	**Carboplatin sub-cohort (*N* = 104)**	**Test of difference between sub-cohorts**
Male		76 (79.2%)	72 (69.2%)	χ^2^ *df*(1), *p* = 0.15
Age	Median (IQR) Mean (SD)	63 (56,68) 61.2 (8.66)	69 (62,74) 67.9 (8.42)	*t*-test, *p* < 0.01
Tumor site	Bladder	77 (80.2%)	76 (73.0%)	χ^2^ *df*(1), *p* = 0.25^d^
	Ureter	11 (11.5%)	19 (18.3%)	
	Renal pelvis	<6	8 (7.7%)	
	Urethra	<6	<6	
TNM at index	T4b,N0,M0	<6	<6	χ^2^ *df*(1), *p* < 0.01^e^
	Tany,N1-3,M0	50 (52.1%)	32 (30.8%)	
	Tany,Nany,M1	42 (43.8%)	70 (67.3%)	
Smoking status	Current smoker	26 (27.1%)	27 (26.0%)	χ^2^ *df*(2), *p* = 0.99^f^
	Ex-smoker	41 (42.7%)	45 (43.3%)	
	Never smoked	9 (9.4%)	9 (8.7%)	
	Non-smoker (history NK)	12 (12.5%)	14 (13.5%)	
	Smoking status NK	8 (8.3%)	9 (8.7%)	
ECOG^a^	0	43 (44.8%)	21 (20.2%)	χ^2^ *df*(1), *p* < 0.001^g^
	1	27 (28.1%)	45 (43.3%)	
	2	<6	18 (17.3%)	
	Not known	25 (26.0%)	20 (19.2%)	
BMI^b^	Mean (SD)	27.60 (4.61)	25.75 (4.69)	*t*-test, *p* < 0.01
	<25	30 (31.3%)	45 (43.3%)	χ^2^ *df*(2), *p* = 0.02^h^
	25–29.9	37 (38.5%)	42 (40.4%)	
	30+	29 (30.2%)	15 (14.4%)	
Site of distant metastases^c^	Lymph (non-pelvic)	27 (64.3%)	40 (57.1%)	χ^2^ *df*(1), *p* = 0.46
	Bone	12 (28.6%)	20 (28.6%)	χ^2^ *df*(1), *p* = 1
	Pulmonary/pleura	11 (26.2%)	22 (31.4%)	χ^2^ *df*(1), *p* = 0.56
	Hepatic	9 (21.4%)	13 (18.6%)	χ^2^ *df*(1), *p* = 0.71

a *ECOG recorded for patients at the first point of receiving treatment with chemotherapy following index date*.

b *Body mass index (kg/m^2^): measures of 25 and above are considered to exceed healthy weight, with 30 being the threshold for obesity in the UK (19). Data was missing for 2 patients in the carboplatin-based sub-cohort*.

c *Categories of distant metastases are not mutually exclusive: patients may have multiple sites. Percentages calculated using denominator of TanyNanyM1*.

d *Comparing bladder and all other sites combined between sub-cohorts*.

e *Comparing TanyNanyM1 and others combined between sub cohorts*.

f *Comparing current smoker, ex-smoker and all others combined between sub-cohorts*.

g *Comparing PS <2 and PS 2+ between sub-cohorts*.

h *Comparing BMI <25, 25–29.9 and 30+*.

**Table 4:** First-line (1L) response rates by type of therapy.

	**All 1L LoT (*n* = 216)**	**1L Cisplatin sub-cohort (*n* = 96)**	**1L Carboplatin sub-cohort (*n* = 104)**	**χ^**2**^*df*(1) comparing sub-cohorts**
Overall Response Rate	51.9%	64.6%	43.3%	
Disease Control Rate	62.5%	75.0%	53.8%	
Complete response	16 (7.4%)	12 (12.5%)	<6	*p* = 0.03
Partial response	96 (44.4%)	50 (52.1%)	41 (39.4%)	*p* = 0.08
Stable disease	23 (10.6%)	10 (10.4%)	11 (10.6%)	*p* = 0.92
Progressive disease	57 (26.4%)	15 (15.6%)	36 (34.6%)	*p* < 0.01
Response missing	24 (11.1%)	9 (9.4%)	12 (11.5%)	
OS Median (95% CI) (months)	16.2 (14.6–18.7)	21.0 (16.2–29.6)	14.6 (11.6–17.4)	
PFS Median (95% CI) (months)	7.6 (6.8–8.7)	10.3 (8.8–12.4)	5.9 (4.6–7.3)	

### Second-line Treatment Patterns

Following treatment with a first-line platinum-based regimen (*n* = 200), 26.5% (*n* = 53) of patients progressed to a second-line treatment. For patients in the cisplatin sub-cohort, 30.2% of patients (*n* = 29) progressed to second-line treatment, compared with 23.1% of patients (*n* = 24) in the carboplatin sub-cohort, but this difference was not statistically significant [χ^2^, *df* (1), *p* = 0.25]. Of the 147 treated with platinum but not progressing to a second-line therapy before study end, 17.7% (*n* = 26) died during treatment and patients on first-line carboplatin were more likely to die during treatment than those on cisplatin [χ^2^, *df* (1), *p* = 0.04].

**Figure 1:**
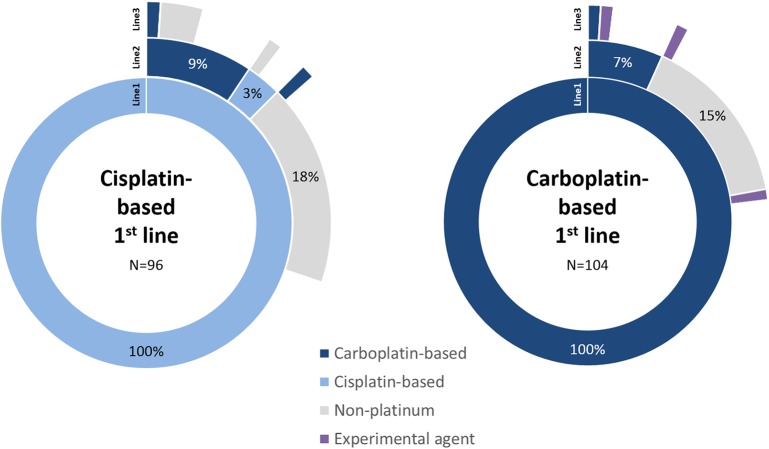
Sequence of line of therapy for patients treated with first-line platinum-based regimens. The first three lines of treatment are shown only, split by cisplatin and carboplatin sub-cohorts. Treatments follow sequentially from inner (Line1) to outer (Line3) segments. Blank segments signify no further treatment recorded.

Non-platinum containing regimens were used second-line for 62.3% (*n* = 33), most commonly single-agent paclitaxel (43.4%; *n* = 23) or gemcitabine (13.2%; *n* = 7). Carboplatin-based regimens were used for 30.2% (*n* = 16) of these patients second-line. The ratio of use of these regimens was similar between the platinum sub-cohorts (Figure 1). For a small proportion of patients treated with first-line cisplatin, a further cisplatin-based regimen was used second-line. Of all patients treated with first-line platinum agents (*n* = 200), 5.5% (*n* = 11) went on to receive at least a third-line of chemotherapy.

The duration of first-line treatment was similar between the two sub-cohorts; median 126 days (IQR: 78,156, range 21–936) for patients receiving cisplatin-based regimens and median 126 days (IQR: 84,143, range 21–828) for those receiving carboplatin. Median duration of second-line chemotherapy was 78 days (IQR: 45,116, range 21–168) for the cisplatin sub-cohort and 120 days (IQR: 100,137, range 21–644) for the carboplatin sub-cohort. The median time between the end of first-line and beginning of second-line was 235 days (IQR: 142,283, range 22–1,102) for the cisplatin sub-cohort and 172 days (IQR: 126,219, range 21–476) for the carboplatin sub-cohort.

No patients receiving non-platinum regimens as first-line treatment received subsequent second-line chemotherapy, but a small number were subsequently treated with palliative radiotherapy (data not shown).

### Survival Outcomes

**Figure 2:**
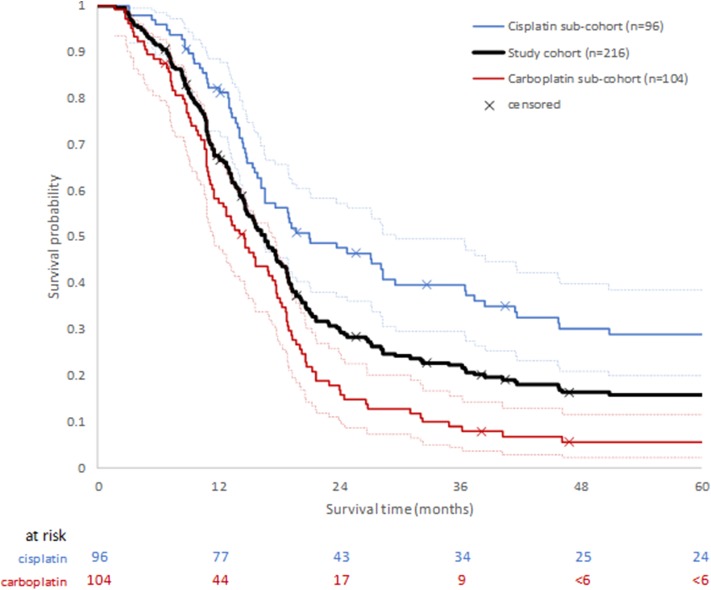
Overall survival following first diagnosis of advanced or metastatic disease for whole study cohort, and by platinum chemotherapy sub-cohort. Ninety five percentage confidence intervals for each platinum sub-cohort are shown by dotted lines.

Median overall survival (mOS) of the study cohort (*n* = 216) was estimated to be 16.2 months (IQR: 10.6, 28.3 months). Where data were stratified by platinum treatment status, the median OS of patients in the cisplatin sub-cohort was 21.0 months (IQR: 13.7, not defined, 95% CI: 16.2–29.6) compared with 14.6 months (IQR: 9.2, 20.3, 95% CI: 11.6–17.4) for the carboplatin sub-cohort, suggesting better survival in the cisplatin treated patients. The 1-year OS post-treatment initiation estimates were 82.2% (95% CI: 72.9–88.5), and 58.3% (95% CI: 48.2–67.2) in the cisplatin and carboplatin sub-cohorts, respectively. The probability of survival remained higher for the cisplatin sub-cohort up to the 5 years for which OS was estimated (Figure 2).

**Figure 3:**
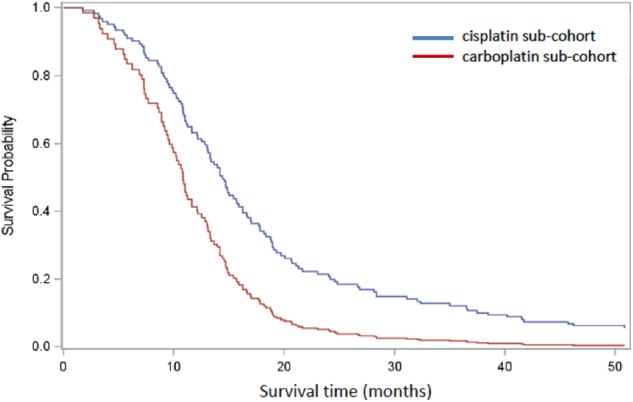
Cox regression analysis comparing cisplatin-based (blue) and carboplatin-based (red) sub-cohorts, adjusted for age, sex, PS, and confirmed metastasis. One hundred and forty five patients remained after patients with missing covariates were excluded.

A Cox PH model was constructed to estimate comparative overall survival for the two platinum groups adjusted for binary categorical variables age (<70 years, 70+yrs), sex (male, female), confirmed metastasis (TNM0, TNM1) and performance score (PS < 2, PS = 2+). These covariates were considered to be predictors of survival and, with the exception of sex, were significantly differentially distributed between the groups. There were 145 patients available for analysis once missing covariates were excluded, and 18.6% (*n* = 27) patients were censored. In the combined model, age (Wald χ^2^ *p* = 0.58) and sex (Wald χ^2^ *p* = 0.74) were not significant contributors but treatment group, PS < 2 and metastatic status (TNM1) were (Wald χ^2^ *p* < 0.05). Once OS was adjusted for differences in age, sex, PS, and metastatic status between the platinum sub-cohorts, HR for survival in patients treated with cisplatin was 0.52 (*p* < 0.01) relative to patients treated with carboplatin (Figure 3).

### Selected Adverse Events Analysis

**Figure 4:**
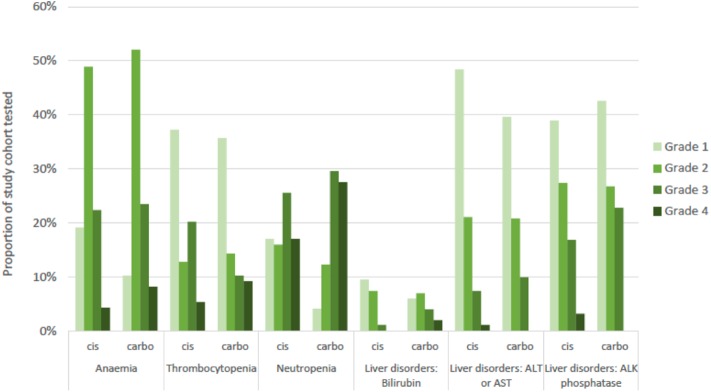
Adverse events recorded for study cohort by common terminology criteria for adverse events (CTCAE) grade of severity and by platinum sub-cohort. Numbers tested: blood disorders (*n* = 192), liver disorders (*n* = 196). Cis, cisplatin sub-cohort; carbo, carboplatin sub-cohort; ALT, alanine aminotransferase; AST, aspartate aminotransferase; ALK, alkaline.

Blood tests to identify hematological and hepatic treatment-related adverse events (AE) were available for 98.0% (*n* = 196) of the platinum-treated sub-cohort. The distribution of events was similar between cisplatin-based and carboplatin-based sub-cohorts, with neutropenia representing the most common grade 3 or 4 AE observed in both sub-cohorts (Figure 4). Severity of recorded events tended to be higher in the carboplatin sub-cohort.

## Discussion

To our knowledge, this is the largest UK-based study looking at real-world outcomes in patients with locally advanced or metastatic urothelial cancer receiving chemotherapy. We examined all patients receiving at least one line of chemotherapy. Just over half (57%) of patients had metastatic (M1) disease at the time of starting chemotherapy. Platinum-based chemotherapy formed the mainstay of first-line treatment, with an almost equal split between those receiving cisplatin- and carboplatin-based regimens (44 vs. 48%). It is of note that a small number of patients (treated before 2010) received single agent paclitaxel or gemcitabine as their first-line of therapy, reflecting a subgroup of patients of older age and poorer PS. Limited data exists to support the use of paclitaxel as a single agent in this setting. A small study of 26 patients reported a response rate of 42% (20), but whether patients deemed unfit for carboplatin would be best served by supportive measures alone, or in the future with a CPI, remains to be established.

Patients receiving a carboplatin-based regimen were older, more likely to have M1 disease and be of poorer PS than those receiving cisplatin-based treatment. Almost one in five patients receiving carboplatin in our cohort were PS2 or 3. We did not document the reasons for choice of carboplatin over cisplatin in good performance patients but presumably this reflects inadequate renal function in most cases. The almost 50% of patients not receiving a cisplatin-containing regimen in our study is in line with previous reports (21, 22).

The activity of platinum-based chemotherapy was confirmed in our study. We estimated an overall response rate of 64.6 and 43.3% in patients receiving cisplatin and carboplatin-based chemotherapy, respectively. This compares with clinical trial reported ORR of 49.4% for cisplatin plus gemcitabine (5) and 41.2% with carboplatin plus gemcitabine (23). The lower response rate associated with carboplatin is consistent with its known inferiority to cisplatin (7). Interpretation of CT reports was performed by an experienced oncologist in our study, but we acknowledge that its retrospective nature may have resulted in an over-estimation of response rates according to RECIST criteria (24).

Overall, median survival of patients in our cohort was 16.2 months. Patients receiving cisplatin-based chemotherapy had a favorable outcome compared to those treated with carboplatin, and this persisted when adjusted for PS, confirmed metastatic status, age and sex. Fisher et al. recently reported median OS of 14.6 months with cisplatin and 11.3 months with carboplatin, in a real-world US setting (25). A 16.1 month median OS was reported in a retrospective German study of patients with stage IV UC treated with chemotherapy (26). Whilst the proportion of M1 patients varied between studies, overall our survival is remarkably similar and highlights the need to improve outcomes for this group of patients.

Only a quarter of patients went on to receive second-line chemotherapy. Just under half of these patients received single-agent weekly paclitaxel. The low uptake of second-line therapy, consistent with previous reports (26), may in part reflect the low response rates and short duration of response associated with these agents (27). Second-line chemotherapy at our center has typically been reserved for fit patients or those with symptomatic disease progression and following careful discussions around risks vs. benefit. Given the recent approvals of both atezolizumab (28) and pembrolizumab (29) following platinum failure, the proportion of patients treated second-line could be expected to increase in future, but none of the reported cohort received these therapies.

We assessed first-line chemotherapy associated adverse event incidence in terms of hematological and hepatic toxicity. Incidence of grade 4 neutropenia associated with cisplatin and carboplatin were 17 and 28%, respectively. This is comparable with rates of 29.9% and 20% in phase III trials of gemcitabine plus cisplatin (5, 30) and 20.3% for gemcitabine plus carboplatin (23). Thus, severe neutropenia rates do not appear to be significantly increased in a real-world setting.

The upper quartile range of age for this cohort was 72–83 years. As the population ages, learning how to optimally assess and manage elderly patients will become increasingly important. We did not collect data on patients who received best supportive care alone and we therefore cannot assess the reasons for this and the demographics of such patients. This remains a critical area for future research (21). There are both strengths and limitations to this study. Extraction of patient data using electronic case note review was enhanced by an experienced medical oncologist who undertook clinical review of unstructured patient notes to maximize data completeness. The number of patients with missing data was minimal for a study of this kind and mostly related to missing PS. The inclusion of adverse events is again not typical and further enhances the dataset. The limitations come from the retrospective nature of the study and the lack of inclusion of patients not receiving systemic treatment. The study design also did not include the collection of surgical procedures. Thus, the influence on survival outcomes of the small number of patients who may have undergone radical cystectomy or radical nephroureterectomy following a response to chemotherapy has not been documented.

In summary, we provide a real-world view of treatment and outcomes in the pre-immunotherapy era for patients with advanced or metastatic UC receiving chemotherapy in a UK setting. The poor outcomes seen in our study reflect those observed both in clinical trials and other recent real-world studies and reinforce the fact that survival has remained static in the pre-immunotherapy era. As CPI become increasingly incorporated into routine clinical care, the data presented here will provide an important benchmark to allow the impact of these and other novel therapeutic strategies to be evaluated in the future.

## Data Availability Statement

The datasets generated for this study are drawn from patient records held by Leeds Teaching Hospitals NHS Trust and will not be made publicly available.

## Ethics Statement

This study was completed with UK Health Research Authority formal approval; as part of this process, the need for written consent of participants and the need for ethics approval were waived.

## Author Contributions

SChe, PG, WS, DS, LA, NV, and GH contributed to the conception and design of the study. SChe and KZ led data collection and enhancement. MT performed the statistical analysis. SJai, SK, SP, AH, JJ, SChi, J-AR, MW, SBu, SBr, SJag, CR, and NV represent members of the bladder cancer multi-disciplinary team and contributed to provision of the data. WS and NV wrote the first draft of the manuscript. All authors contributed to manuscript revision, read, and approved the submitted version.

### Conflict of Interest

REAL Oncology is a collaboration between Leeds Cancer Centre (LCC), the University of Leeds and IQVIA™. Commercial clients of IQVIA include Janssen Pharmaceuticals, which funded the work this paper is based on. REAL Oncology retains all operational, scientific, and communications controls. WS, MT, and PG are employees of IQVIA. DS and LA are employees of Janssen. The remaining authors declare that the research was conducted in the absence of any commercial or financial relationships that could be construed as a potential conflict of interest.
